# Xenograft for anterior cruciate ligament reconstruction was associated with high graft processing infection

**DOI:** 10.1186/s40634-020-00292-0

**Published:** 2020-10-07

**Authors:** Willem Van Der Merwe, Martin Lind, Peter Faunø, Kees Van Egmond, Stefano Zaffagnini, Maurilio Marcacci, Ramon Cugat, Rene Verdonk, Enrique Ibañez, Pedro Guillen, Giulio Maria Marcheggiani Muccioli

**Affiliations:** 1 Sport Science Institute of S. Africa, Cape Town, South Africa; 2 Aarhus University Hospital, Aarhus, Denmark; 3 Dept. of Orthopaedic Surgery, Isala Klinieken, Zwolle, Netherlands; 4 IRCCS Istituto Ortopedici Rizzoli, University of Bologna, Lab. Biomeccanica - Via di Barbiano, 1/10, 40137 Bologna, Italy; 5 IRCCS Humanitas University, Milano / former Istituto Ortopedici Rizzoli, University of Bologna, II Clinica Ortopedica, Bologna, Italy; 6 Hospital Quiron, Artoscopia GC, Barcelona, Spain; 7 Dept. of Orthopaedic Surgery & Traumatology, Gent Univ. Hospital, Ghent, Belgium; 8 Clinica Cemtro, Orthopaedic Surgery & Traumatology, Madrid, Spain

**Keywords:** Anterior cruciate ligament, Reconstruction, Biomaterial, Tissue engineering, Allografts, Xenografts

## Abstract

**Purpose:**

To evaluate clinical ad radiological outcomes of anterior cruciate ligament (ACL) reconstruction with an immunochemically modified porcine patellar tendon xenograft controlled against human Achilles tendon allograft at 24-month minimum follow-up.

**Methods:**

66 patients undergoing arthroscopic ACL reconstruction were randomized into 2 groups: 34 allografts and 32 xenografts treated to attenuate the host immune response. Follow-up was 24-month minimum. Anterior knee stability was measured as KT − 1000 side-to-side laxity difference (respect to the contralateral healthy knee). Functional performance was assessed by one-legged hop test. Objective manual pivot-shift test and subjective (IKDC, Tegner and SF-36) outcomes were collected. MRI and standard X-Ray were performed.

**Results:**

61 subjects (32 allograft, 29 xenograft) were evaluated at 12 and 24 months. Six of the subjects in xenograft group (20.6%) got an infection attributed to a water-based pathogen graft contamination in processing.

Intention-to-treat analysis (using the last observation carried forward imputation method) revealed higher KT − 1000 laxity in xenograft group at 24-month follow-up (*P* = .042). Also pivot-shift was higher in xenograft group at 12-month (*P* = .015) and 24-month follow-up (*P* = .038).

Per-protocol analysis (missing/contaminated subjects excluded) did not revealed clinical differences between groups.

Tibial tunnel widening in the allograft group was low, whereas xenograft tunnel widening was within the expected range of 20–35% as reported in the literature.

No immunological reactivity was associated to xenograft group.

**Conclusions:**

High infection rate (20.6%) was reported in xenograft group. Both groups of patients achieved comparable clinical outcomes if missing/contaminated subjects are excluded. Improved harvesting/processing treatments in future studies using xenografts for ACL reconstruction are needed to reduce infection rate, otherwise xenograft should not be used in ACL reconstruction.

**Level of evidence:**

Multicenter and double-blinded Randomized Controlled Clinical Trial, Level I.

## Introduction

The anterior cruciate ligament (ACL) is the key stabilizer of the knee joint and is frequently injured in athletic activities. Each year, an estimated 80,000 to more than 250,000 ACL injuries occur, many in young athletes 15 to 25 years of age [9]. Surgical techniques currently shows excellent results with either the use of the patient’s own tissue to reconstruct the ACL (autograft) or, less frequently, cadaveric tissue graft (allograft).

The concept of using xenograft tissue, defined as graft tissue from one species and destined for implantation in an unlike species, was introduced in 90’s. The potential advantages of xenograft use in ACL reconstruction could be to overcome the safety, mechanical and quality concerns and availability problems of allograft tissue [31]. A potential reduction of the costs could also be reached using xenograft instead of allograft.

The cause of immunologic rejection when transplanting animal tissues into humans was identified in multiple studies as a reaction to the α-Gal epitope (Galα1-3Galβ1-4GlcNAc-R) present on cells and extracellular matrix from animal, but not human, tissues [1, 13, 23, 24, 30].

The positive results and the safety profile reported in the animal model were replicated in the first clinical trial involving 10 humans, who confirmed the safety of immunochemically modified porcine patellar tendon xenograft. The xenograft was processed with the glycosidase enzyme, α-galactosidase, effectively attenuating host to graft immune recognition by α-Gal epitope cleavage [23].

The aims of the current study was to evaluate clinical ad radiological outcomes of ACL reconstruction with the same immunochemically modified porcine patellar tendon xenograft device controlled against human allograft at 24-month follow-up.

The hypothesis of the present study was that the de-antigenated porcine patellar tendon xenograft device would perform as well as the allograft tendon as measured by objective and subjective clinical outcomes at 24-month minimum follow-up.

## Material and methods

This prospective, randomized, double-blinded clinical study was carried out at seven institutions in the European Union and South Africa. Investigators were un-blinded at 24 months.

From January 2011 to April 2012, 66 patients with acute or chronic ACL ruptures underwent ACL reconstruction. Patients were randomized 1:1 to receive either allograft or xenograft. Prior to surgery, all subjects were enrolled into the study after providing an informed consent.

The inclusion criteria were: (1) acute or chronic primary ruptured ACL as documented by i) physical exam with positive Lachman’s Test, or ii) ≥ 5-mm side to side difference by KT-1000, or iii) confirmatory MRI; (2) 18–60 years of age; (3) simple ACL insufficiency with no concurrent multi-axial or multi-ligamentous instabilities or knee dislocations; (4) pre-injury Tegner Score < 8; and (5) osteoarthritis < Grade IV on the ICRS scale. The exclusion criteria were the following: (1) acute inflammation or effusion of the injured knee; (2) meniscectomy > 50%; (3) any etiologies that may affect rehabilitation; (4) inflammatory arthritis; (5) active or latent infection; (6) previous allograft or autograft procedure to either knee; (7) BMI > 40; and (8) subjects planning on returning to competitive sports within 9 months post-surgery; (9) any kind of surgery in the contralateral knee.

### Preparation of grafts

Allograft bone/tendon (Achilles) grafts suitable for ACL reconstruction were obtained from regional or hospital based tissue banks in compliance with European Association of Tissue Bank standards, from donors aged 18 to 65 years, and were representative of the standard of care for allografts for each clinical site. Allografts were either aseptically processed or exposed to low dose irradiation. On the day of the operation, the graft was thawed in sterile physiologic fluid, with or without antibiotic, at room temperature before implantation. Grafts were kept hydrated prior to implantation.

Xenografts were processed as previously described [22, 25] and were supplied sterile and frozen by the study sponsor (XXX, XXX, XX, XXX). In brief, porcine bone patellar-tendon-bone constructs from skeletally mature pigs were harvested and procured from a breeder and abattoir compliant with ISO 22442 requirements for medical implantation grade materials. Grafts were precision machined with a cylindrical proximal bone plug block (10 mm diameter, 25 mm length), uniform mid-tendon width (10 mm) and minimal thickness (5 mm), with a distal accessory bone plug (5 mm thick, 8 mm length). After decellularization and rinsing, grafts were incubated with α-galactosidase enzyme to cleave α-gal epitopes. After further rinsing, grafts were subjected to low-level glutaraldehyde cross-linking and end-capping, which stabilizes the collagenous structure and attenuates non-specific porcine epitope recognition [16, 22, 25]. The final processing stage includes copious rinsing of the devices to remove any residual chemicals. The devices were then packaged, frozen, and terminally sterilized with an electron beam dose of approximately 18 kGy. All processing was performed under manufacturing control by the study sponsor. Peri-operative handling was identical to allografts.

### Surgical technique

A routine arthroscopy of the entire knee joint was performed, followed by ACL reconstruction as described below and consistent with standard practice.

Through direct visualization at the time of surgery, the investigators confirmed that the pre-operative screening evaluations were correct and that the knee met the study inclusion and exclusion criteria. Any associated meniscal or articular cartilage damage was repaired or resected. For the creation of the femoral tunnel, one site used the transtibial technique; the remainder of the sites used anteromedial portal anatomic technique. The tibial tunnel (9 mm) was placed to position the device anatomically within the fibers of the original ACL and without roof impingement. The femoral tunnel (9 mm) was placed within the original ACL insertion and in the posterior one-quarter of the femoral condyle (only 2 mm of bone on the posterior wall of the femoral tunnel remained). The device was prepared to fit tunnels and pre-tensioned (70 N of pre-tension force was applied for 5 min). Therefore, it was pulled into place and fixed with titanium, PEEK or PLLA interference screws distributed by multiple suppliers as CE Marked devices (see Additional file 1 for details). The knee was moved through a full range of motion to ensure that there was no impingement and was manually tested for stability.

### Rehabilitation

All the patients underwent the same rehabilitation protocol, [29] which began at the time of the initial diagnosis. Patients used a post-operative extension brace for 2 weeks. Rehabilitation followed a stage-gated protocol with incremental weight bearing and closed to open chain exercise in the 0 to 6 weeks post-operative interval. Gradual return to sports was allowed after 6 months, with return to competitive sports after 9 months.

### Objective clinical evaluation

Objective clinical evaluation of patients was performed both pre-operatively and at 12- and 24-month follow-up visits. Functional performance was assessed also at 6-month follow-up.

Objective clinical evaluation consisted of instrumented Lachman test and manual pivot-shift test. Graft efficacy was determined by instrumented knee laxity measurement. Efficacy success was defined by a KT-1000 manual-maximum Lachman test (at 25° of knee flexion) side-to-side difference value ≤5 mm (side to side difference of treated vs. non- treated knee). Success by Lachman’s test required either a Grade A (− 1 to 2 mm) or B (3 to 5 mm) with a “firm” anterior endpoint [10]. Success by pivot shift required either Grade A (Equal) or B (Glide) [14]. In cases where data was missing in any of the measures, that measure was treated as a failure at that time point.

Graft functional performance success was defined via the one-legged hop assessment. A subject was considered successful if functional performance of the index knee achieved ≥90% of the functional performance of the contralateral (unoperated) knee.

Objective laxity and functional performance assessments at each clinical site were performed by the same blinded assessor to minimize inter-operator variability.

Graft safety evaluations tabulate serious adverse events on a group comparison basis. Adverse events and risk assessment were reviewed and reported by an independent medical monitor.

### Subjective clinical evaluations

Subjective evaluation of patients both pre-operatively and at 12- and 24-month follow-up visits used the pain questionnaire of International Knee Documentation Committee (IKDC) Subjective Knee Evaluation Form, Tegner Activity Index, and SF-36 Health Survey [3, 15, 26].

### Radiological evaluation

Radiographic (standard non-weight bearing antero-posterior and medial-lateral projections) and 1,5 Tesla magnetic resonance imaging (MRI) assessments were performed at 1 week, 6 months, 12 months, and 24 months and were reviewed by a central, independent, blinded radiologist. Maximum graft cross-sectional area (CSA), bone tunnel diameter and bone edema diameter were measured on MRI scans by means of KODAK CARESTREAM PACS tools. With the same measuring tools we determined the increase in bone tunnels on X-rays. Graft signal, cartilage and meniscal findings were qualitatively evaluated by MRI.

### Serological and immunologic evaluation

Serological testing: serological assessments taken at baseline and at all follow-up visits included: blood chemistries, cell blood count (CBC), inflammation markers of C-reactive protein and erythrocyte sedimentation rate (ESR). Samples were analyzed and reported by an independent blinded laboratory.

Anti-Gal and anti-non Gal antibody titers were measured to determine if an acute rejection of the graft or a graft versus host response occurred.

Anti-Gal testing: The anti-Gal antibody response indicates the antibody response to the galactosyl epitope. The anti-Gal antibody response was determined by performing enzyme-linked immunosorbent assay (ELISA) with wells coated with synthetic α-gal epitopes linked to bovine serum albumin (10 micrograms / ml) [22]. Bovine serum albumin (1%) in PBS was placed in the wells for 2 h in order to block subsequent nonspecific antibody binding. Samples underwent serial two-fold dilutions with a starting dilution of 1:10. After incubating the plates at room temperature for 2 h, plates were washed, and a rabbit anti-human IgG antibody was added for 1 h. After an additional wash, the color reaction was developed with o-phenylenediamine substrate and absorbance at 492 nm was determined. The extent of anti-Gal antibody response was represented by the fold increase in the antibody response in the post-implantation serum in comparison to the baseline pre-implantation binding curve in each individual patient. The antibody activity (titer) is presented as the reciprocal of serum dilution at 50% maximum binding.

Anti-Non Gal testing: the anti-non Gal response indicates the antibody response to porcine epitopes other than the galactosyl epitope. The anti-non Gal antibody response was determined by performing an ELISA with a homogenate of porcine tendon fragments (10 mg/ml) in saline as solid phase antigen [22]. The drying of the tendon fragments in ELISA wells results in their firm adhesion to the wells. Bovine serum albumin (1%) in PBS was placed in the wells for 2 h in order to block subsequent nonspecific antibody binding. To prevent detection of anti-Gal antibodies in this ELISA, the serum was first depleted of anti-Gal antibodies by adsorption on glutaraldehyde-fixed rabbit red blood cells. Samples underwent serial two-fold dilutions with a starting dilution of 1:10. Plates were then incubated for 2 h at room temperature, followed by washing and subsequent addition of a rabbit anti-human IgG antibody for a 1 h incubation. After an additional wash, the color reaction was developed with o-phenylenediamine and absorbance at 492 nm was determined. The extent of anti-non Gal antibody response was represented by the fold increase in the antibody response in the post-implantation serum in comparison to the baseline pre-implantation binding curve in each individual patient. The antibody activity (titer) is presented as the reciprocal of serum dilution at 50% maximum binding.

This study obtained the approval by the local from Institutional Review Board (IRB) of each center and national competent authority, where applicable.

### Statistical analysis

This was a two-arm prospective randomized trial with a 1:1 randomization ratio. The randomization was generated per institution using small block size (< 8). Sample size was determined prospectively based on α = 0.05, power = 0.80, and an expected difference between proportion of successful grafts in the treatment groups of 0.10. Following these hypotheses, the necessary sample size in each of the treatment groups was estimated at 28 subjects. Continuous variables are summarized using mean and standard deviation. Categorical variables are summarized by categories using percentages. Baseline variables are described by treatment group and compared using a Student or Wilcoxon rank sum test for continuous variables and Chi square or Fisher exact test for categorical variables. 90% confidence intervals for the efficacy and performance endpoints were approximated using the Agresti-Coull interval.

An intention-to-treat (ITT) analysis was performed including missing/contaminated graft subjects and using the Last Observation Carried Forward (LOCF) imputation method. Another per-protocol analysis was performed including only subjects who completed the study without any major protocol violations (missing/contaminated graft subjects excluded).

All analyses were performed by a third party clinical statistician. Significance was set at *P* < .05.

## Results

### Patients demographics

**Fig. 1:**
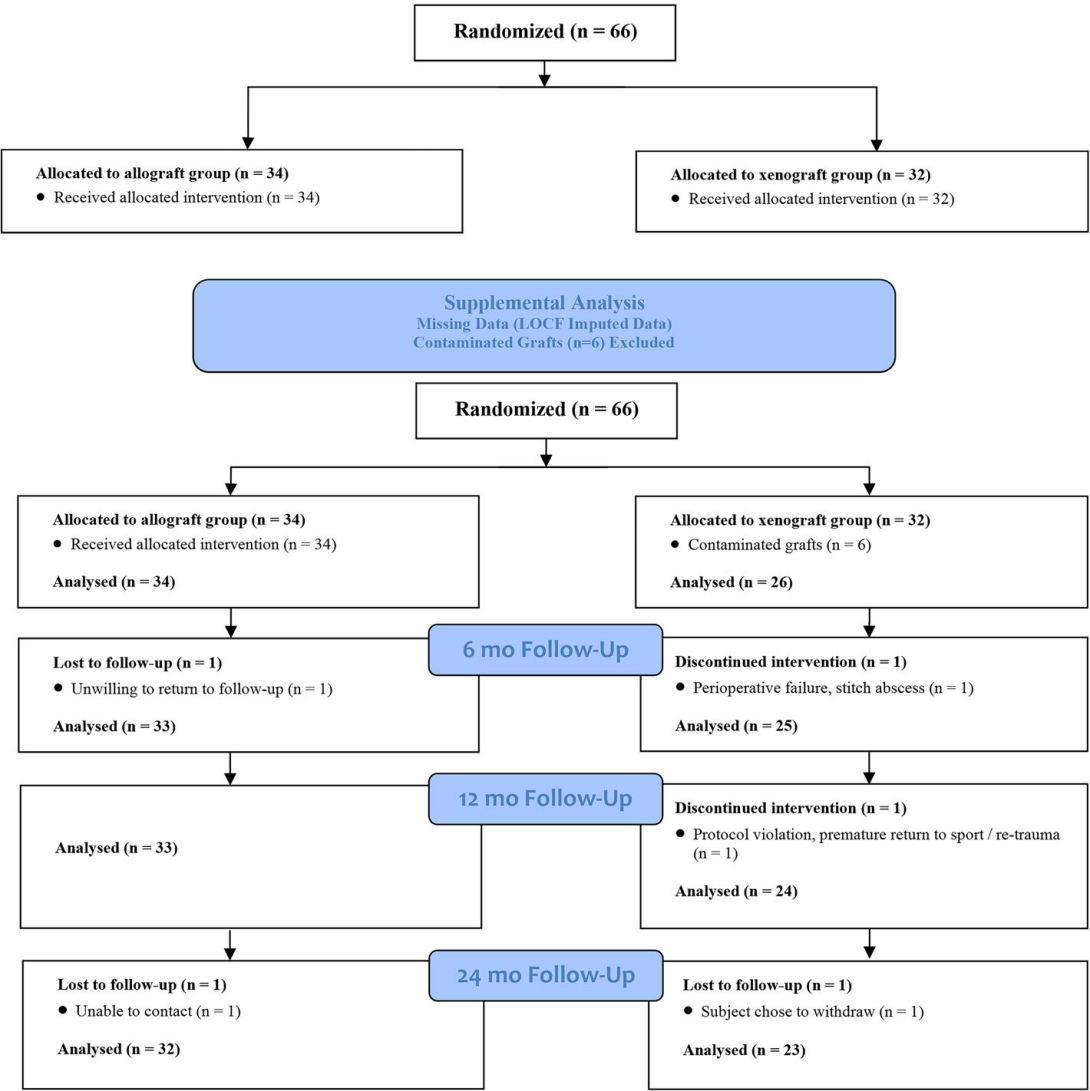
Flow-chart of inclusion, exclusion and analysis process into this randomized controlled clinical trial

Of the 66 subjects entered into the study, 61 (*N* = 32 in the allograft group, and 29 in the xenograft group) successfully completed the assigned 24-month minimum follow-up protocol. Five were excluded from the study as follows (Fig. 1): 2 in the allograft group (both lost to follow-up) and 3 in the xenograft group (1 lost to perioperative failure – stitch abscess; 1 lost to protocol violation – premature return to sport/re-trauma; and 1 elected to withdraw).

Patient demographics and characteristics are outlined in Table 1. Sporting activities were the main cause of injury in the patients. There were no statistically significant differences in demographics between the two groups.

**Table 1:** Demographic Data and Characteristics of Patients (*N* = 66)

	Allo (***n*** = 34)	Xeno (***n*** = 32)	***P***-value
**Age at Surgery (mean ± sd, yrs)**	30.5 ± 9.6 (18–52)	31.6 ± 9.1 (18–52)	0.517
**Gender N (%)**	22 (64.7%)M | 12 (35.3%)F	27 (84.4%)M | 5 (15.6%)F	0.068
**Side N (%)**	13 (38.2%)R | 21 (61.8%)L	15 (46.9%)R | 17 (53.1%)L	0.430
**Time from injury to surgery (mo)**	10.7 ± 18.7	13.8 ± 21.9	0.549
**Follow-up interval (mo)**	25.8 ± 1.8 (24.0–32.7)	25.8 ± 8.7 (2.1–38.0)	0.098
**Mechanism of initial injury N (%)**			0.891
**Sports**	24 (70.6%)	26 (81.3%)	
**ADL**	4 (11.8%)	3 (9.4%)	
**Motorbike**	3 (8.8%)	1 (3.1%)	
**Work**	1 (2.9%)	1 (3.1%)	
**Other**	2 (5.9%)	1 (3.1%)	

Six of the subjects in xenograft group (20.6%) got a deep infection attributed to a water-based pathogen graft contamination in that occurred during the graft processing. These infections developed in four different centers (center no. 2,4,7,8) and was related to the first batch of xenografts. In the following released batch the water contamination was corrected and no infection occurred in the patients impanted with this xenografts. These subjects were considered failures and were included into analysis after graft removal and arthroscopic debridement surgery plus antibiotic therapy (see also Table 2).

**Table 2:** Serious Adverse Event Data Summary

Subject	Study Group	Number of SAEs	Description
2F	Xenograft	1	• Surgical site stitch abscess
2O^a^	Xenograft	1	• Device infection requiring graft removal
2AE	Xenograft	1	• Persistent effusion and pain
4D^a^	Xenograft	2	• Post-op septic arthritis • Persistent effusion requiring graft removal
4F^a^	Xenograft	1	• Synovitis, tibial tunnel osteomyelitis requiring graft removal
7A^a^	Xenograft	4	• Persistent inflammation and surgical site fistula • Tibial screw removal • Tibial tunnel osteomyelitis • Persistent inflammation, surgical site fistula requiring graft removal
7D	Xenograft	1	• Persistent pain
7E	Xenograft	1	• Traumatic ACL re-rupture
7I	Allograft	1	• Myocardial infarction
7 N^a^	Xenograft	3	• Synovitis • Persistent inflammation, surgical site fistula requiring graft removal • Surgical site septic arthritis, post graft removal
8B^a^	Xenograft	1	• Traumatic ACL re-rupture

NOTE: ^a^ indicates subject received device associated with water-based pathogen

### Objective clinical outcomes

Objective knee laxity scores are presented in Table 3. ITT analysis (using the LOCF imputation method) revealed significant higher KT-1000 knee laxity values (at manual-maximum force) in xenograft group at 24-month follow-up (*P* = .042). Also pivot-shift was significantly higher in xenograft group at 12-month (*P* = .015) and 24-month follow-up (*P* = .038) (Table 3). Per-protocol analysis (missing/contaminated graft subjects excluded) did not revealed significant objective laxity differences between groups (Table 4).

**Table 3:** Objective Knee Laxity Evaluation - Missing/Contaminated Graft Subjects Included (LOCF)

	Preoperative			12 Months			24 Months		
	**Allograft**	**Xenograft**	***P***	**Allograft**	**Xenograft**	***P***	**Allograft**	**Xenograft**	***P***
**Pivot Shift n (%)**									
**Equal**	4 (11.8)	2 (6.7)	.932 (Fisher)	32 (94.1)	23 (71.9)	.015* (Fisher)	31 (91.2)	22 (68.8)	.038* (Fisher)
**Glide**	12 (35.3)	11 (36.7)		2 (5.9)	9 (28.1)		3 (8.8)	9 (28.1)	
**Clunk**	17 (50.0)	16 (53.3)		0 (0)	0 (0)		0 (0)	1 (3.1)	
**Gross**	1 (2.9)	1 (3.3)		0 (0)	0 (0)		0 (0)	0 (0)	
**total n**	34	30		34	32		34	32	
**Lachman’s Test** **KT-1000 Man-Max n (%)**									
**-1 to 2mm**	0 (0)	1 (3.1)	.970 (Fisher)	30 (88.2)	22 (68.8)	.092 (Fisher)	31 (91.2)	22 (68.8)	.042* (Fisher)
**3 to 5 mm**	11 (32.4)	10 (31.3)		4 (11.8)	7 (21.9)		3 (8.8)	6 (18.8)	
**6 to 10 mm**	18 (52.9)	16 (50.0)		0 (0)	3 (9.4)		0 (0)	4 (12.5)	
**> 10 mm**	5 (14.7)	5 (15.6)		0 (0)	0 (0)		0 (0)	0 (0)	
**total n**	34	32		34	32		34	32	

*p < 0.05

**Table 4:** Objective Knee Laxity Evaluation - Missing/Contaminated Graft Subjects Excluded

	Preoperative			12 Months			24 Months		
	**Allograft**	**Xenograft**	***P***	**Allograft**	**Xenograft**	***P***	**Allograft**	**Xenograft**	***P***
**Pivot Shift n (%)**									
**Equal**	4 (11.8)	1 (4.2)	.892 (Fisher)	31 (3.9)	18 (75.0)	.059 (Fisher)	29 (90.6)	17 (73.9)	.143 (Fisher)
**Glide**	12 (35.3)	9 (37.5)		2 (6.1)	6 (25.0)		3 (9.4)	6 (26.1)	
**Clunk**	17 (50.0)	13 (54.2)		0 (0)	0 (0)		0 (0)	0 (0)	
**Gross**	1 (2.9)	1 (4.2)		0 (0)	0 (0)		0 (0)	0 (0)	
**total n**	34	24		33	24		32	23	
**Lachman’s Test** **KT-1000 Man-Max n (%)**									
**-1 to 2 mm**	0 (0)	1 (3.8)	.771 (Fisher)	29 (87.9)	17 (70.8)	.215 (Fisher)	29 (90.6)	17 (73.9)	.173 (Fisher)
**3 to 5 mm**	11 (32.4)	8 (30.8)		4 (12.1)	6 (25.0)		3 (9.4)	5 (21.7)	
**6 to 10 mm**	18 (52.9)	12 (46.2)		0 (0)	1 (4.2)		0 (0)	1 (4.3)	
**> 10 mm**	5 (14.7)	5 (19.2)		0 (0)	0 (0)		0 (0)	0 (0)	
**total n**	34	26		32	24		31	23	

*p < 0.05

**Fig. 2:**
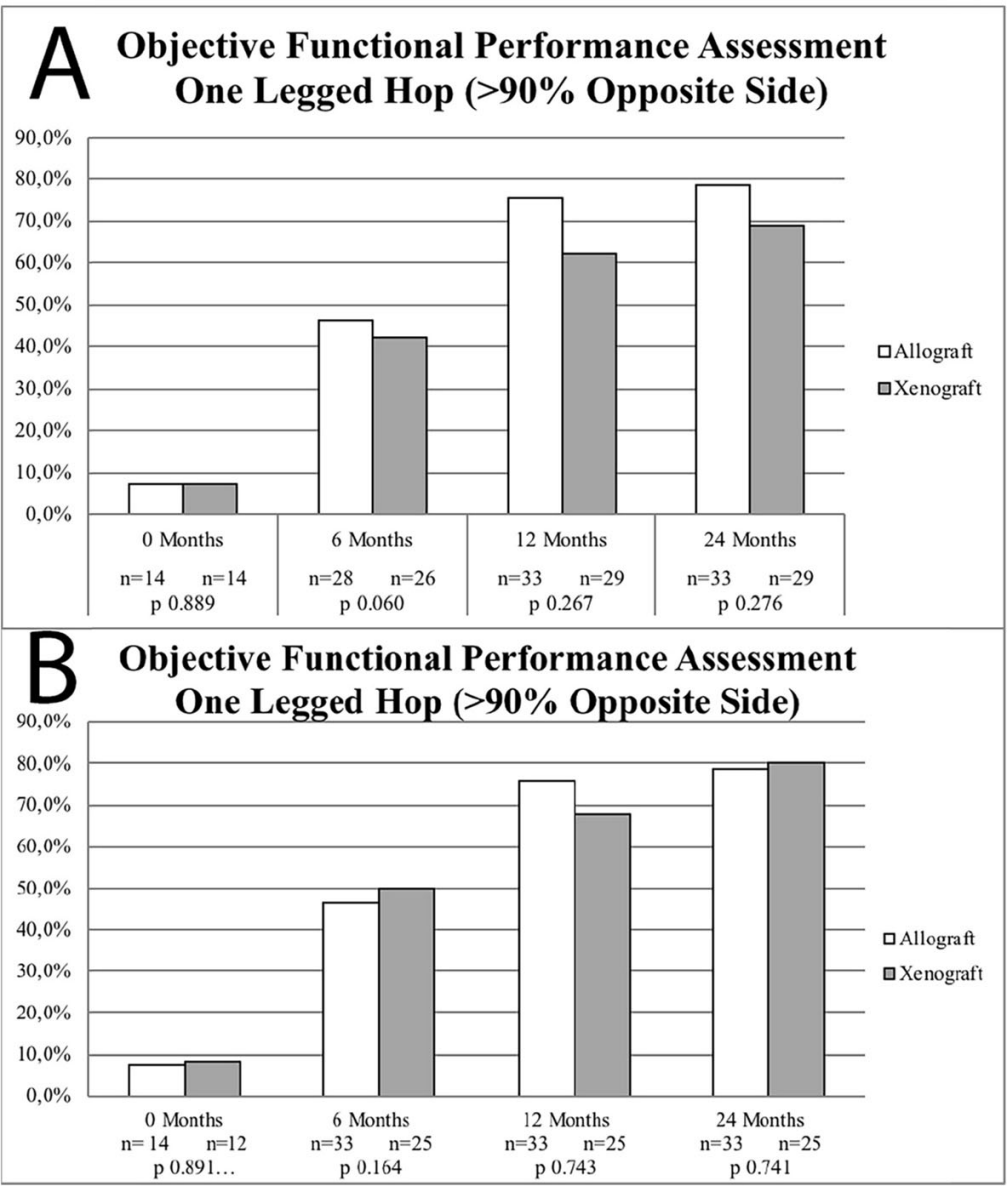
Graft Functional Performance Assessment (One Leg Hop Test) - **a**, Missing/Contaminated Graft Subjects Included (LOCF); **b**, Missing/Contaminated Graft Subjects Excluded. No statistically significant differences were reported between study groups in both analysis (intention-to-treat vs. per protocol)

Functional performance assessment is presented in Fig. 2. Both ITT analysis (using the LOCF imputation method, Fig. 2a) and per-protocol analysis (missing/contaminated graft subjects excluded, Fig. 2b) did not revealed significant functional performance differences between groups.

### Subjective clinical outcomes

**Fig. 3:**
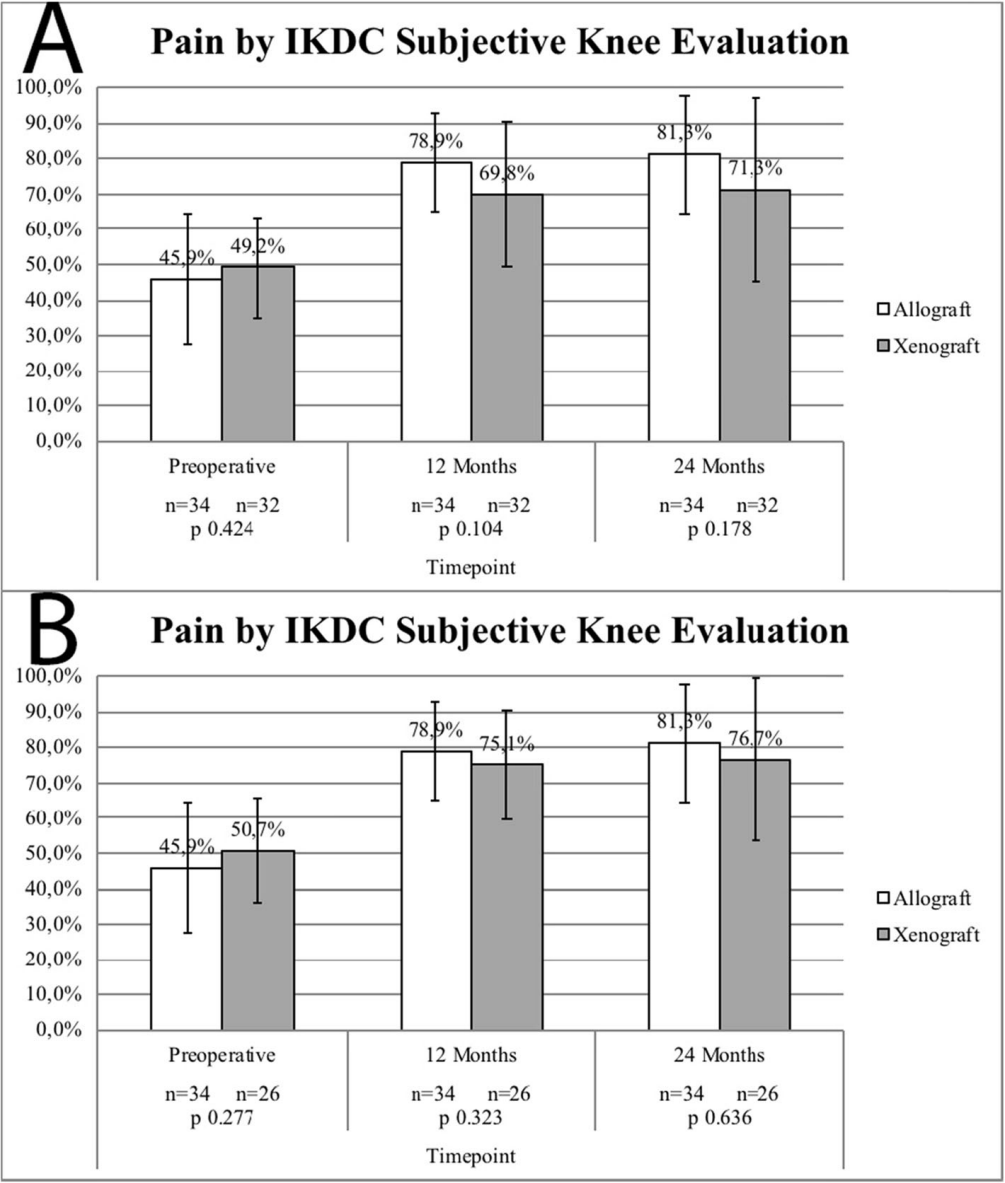
Pain by IKDC Subjective Evaluation Scores - **a**, Missing/Contaminated Graft Subjects Included (LOCF); **b**, Missing/Contaminated Graft Subjects Excluded. No statistically significant differences were reported between study groups in both analysis (intention-to-treat vs. per protocol)

**Fig. 4:**
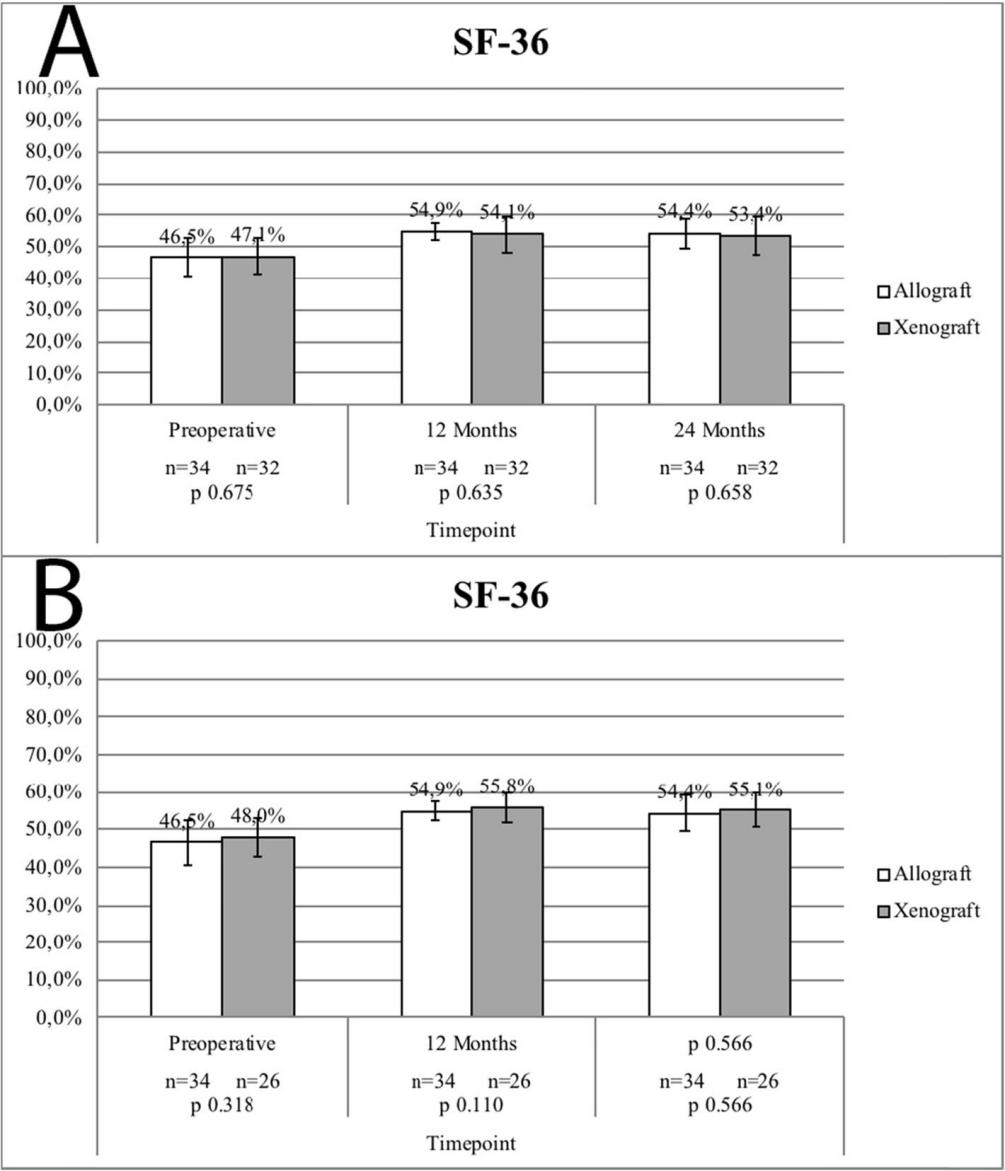
SF-36 Scores - **a**, Missing/Contaminated Graft Subjects Included (LOCF); **b**, Missing/Contaminated Graft Subjects Excluded. No statistically significant differences were reported between study groups in both analysis (intention-to-treat vs. per protocol)

**Fig. 5:**
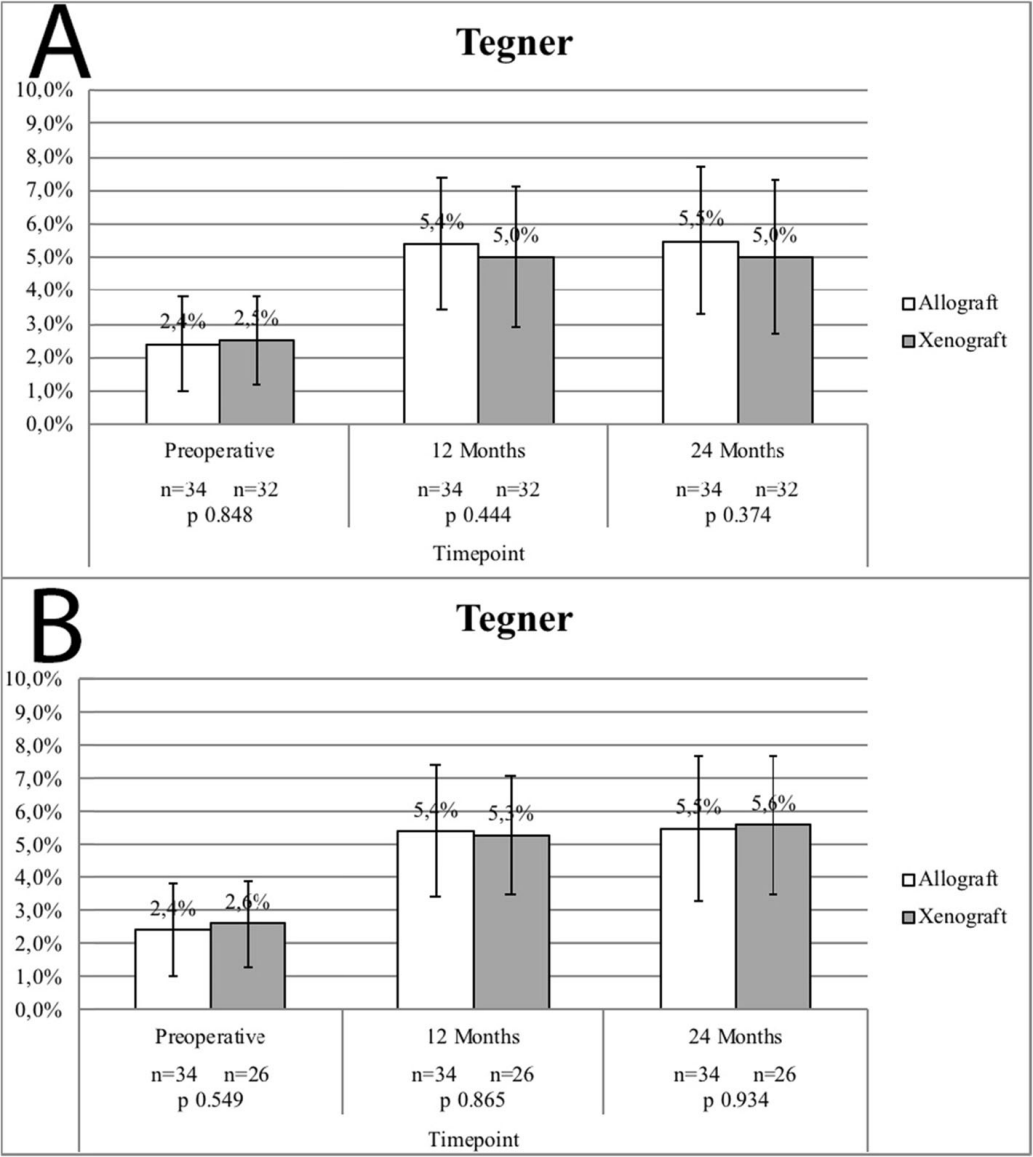
Tegner Scores - **a**, Missing/Contaminated Graft Subjects Included (LOCF); **b**, Missing/Contaminated Graft Subjects Excluded. No statistically significant differences were reported between study groups in both analysis (intention-to-treat vs. per protocol)

Subjective pain by IKDC, SF-36 and Tegner assessments are presented in Figs. 3, 4 and 5 respectively. Both ITT analysis (using the LOCF imputation method, Fig. 3a, 4a e 5a) and per-protocol analysis (missing/contaminated graft subjects excluded, Fig. 3b, 4b e 5b) did not revealed significant subjective clinical outcomes differences between groups.

### Radiologic results

Femoral and tibial bone tunnel dimensions, as measured on MRI, were equivalent at one week post-surgically. Significant differences in graft cross-sectional area and tunnel dimensions between groups were detected at both the 12 and 24-month time points both considering ITT analysis (using the LOCF imputation method, Table 5) and per-protocol analysis (missing/contaminated graft subjects excluded, Table 6).

**Table 5:** MRI Evaluation (Cross-Sectional Area and Tunnel Dimensions) - Missing/Contaminated Graft Subjects Included (LOCF)

	1 Week				12 Months				24 Months		
	Allo	Xeno	p-value		Allo	Xeno	p-value		Allo	Xeno	p-value
CSA_max_	74.1 ± 28.6	69.1 ± 24.6	0.459		106.9 ± 49.9	120.9 ± 66.9	0.447		92.0 ± 32.9	112.3 ± 51.1	0.044*
FT–AP	10.2 ± 1.3	10.0 ± 0.9	0.606		9.6 ± 1.7	13.6 ± 2.3	< 0.001**		10.2 ± 2.2	11.9 ± 2.5	0.085
FT–ML	10.0 ± 1.2	9.3 ± 0.7	0.067		11.2 ± 2.3	14.5 ± 3.2	0.004*		9.6 ± 2.0	13.0 ± 1.9	< 0.001**
TT–AP	9.7 ± 1.6	9.0 ± 2.0	0.053		10.3 ± 2.7	12.6 ± 2.5	< 0.001**		11.4 ± 2.5	13.3 ± 2.1	0.009*
TT–ML	9.4 ± 1.6	9.7 ± 1.7	0.408		10.3 ± 2.1	13.0 ± 2.5	< 0.001**		10.1 ± 2.0	12.1 ± 1.7	< 0.001**

**Table 6:** MRI Evaluation (Cross-Sectional Area and Tunnel Dimensions) - Missing/Contaminated Graft Subjects Excluded

	1 Week				12 Months				24 Months		
	Allo	Xeno	p-value		Allo	Xeno	p-value		Allo	Xeno	p-value
CSA_max_	74.1 ± 28.6	68.4 ± 21.2	0.403		106.9 ± 49.9	121.9 ± 70.4	0.529		92.0 ± 32.9	113.8 ± 51.9	0.036*
FT–AP	10.2 ± 1.3	9.9 ± 1.0	0.571		9.6 ± 1.7	13.6 ± 2.2	< 0.001**		10.2 ± 2.2	11.8 ± 2.6	0.122
FT–ML	10.0 ± 1.2	9.3 ± 0.8	0.096		11.2 ± 2.3	14.1 ± 3.2	0.011*		9.6 ± 2.0	13.1 ± 2.0	< 0.001**
TT–AP	9.7 ± 1.6	9.1 ± 2.2	0.095		10.3 ± 2.7	12.2 ± 1.8	< 0.001**		11.4 ± 2.5	13.3 ± 2.2	0.013*
TT–ML	9.4 ± 1.6	9.8 ± 1.7	0.339		10.3 ± 2.1	12.5 ± 2.1	< 0.001**		10.1 ± 2.0	12.1 ± 1.7	< 0.001**

*p < 0.05; **p < 0.001; CSA_max_, Maximum graft cross-sectional area in mm^2^;FT–AP, Femoral tunnel anterior-posterior in mm; FT–ML, Femoral tunnel medial-lateral in mm; TT–AP, Tibial tunnel anterior-posterior in mm; TT–ML, Tibial tunnel medial-lateral in mm

To better understand the bone tunnel widening seen by MRI, 1 week and 12 month X-rays of femoral and tibial bone tunnels were assessed using a quantitative imaging technique and compared between groups (with an intention-to-treat analysis, using the LOCF imputation method) and to the expected extent of tunnel widening (20–35% increase in diameter at 12 months). Tibial tunnel widening in the allograft group was exceptionally low, whereas xenograft tunnel widening was within the range of 20–35% (Table 7). Femoral tunnel widening was low in both groups at 12 months, and no significant site-to-site differences were noted (Table 7).

**Table 7:** X-ray Evaluation of Tunnel Changes (Increase in Tunnel Diameter, 12 Month versus 7 Day) - Missing/Contaminated Graft Subjects Included (LOCF)

		Tibial				Femoral		
		Allo	Xeno			Allo	Xeno	
AP		5.69%	28.86%			0.94%	11.08%	
ML		7.10%	28.00%			0.00%	1.02%	

*AP* Anterior-Posterior, *ML* Medial-Lateral

Bone edema, as measured on MRI, were equivalent at one week, 12 months, and 24 months post-surgically, with both groups trending toward no edema on PDFS images (Additional file 2).

Cartilage and meniscal findings (meniscal tears, loose bodies, changes in articular cartilage injury, and other) were assessed by MRI and tallied at one week, 12 months, and 24 months-post-surgically (Additional file 2). Both ITT analysis (using the LOCF imputation method, Additional file 2A) and per-protocol analysis (missing/contaminated graft subjects excluded, Additional file 2B) did not revealed significant differences between groups in any of these assessments at any of the three time points.

### Immunologic results

**Fig. 6:**
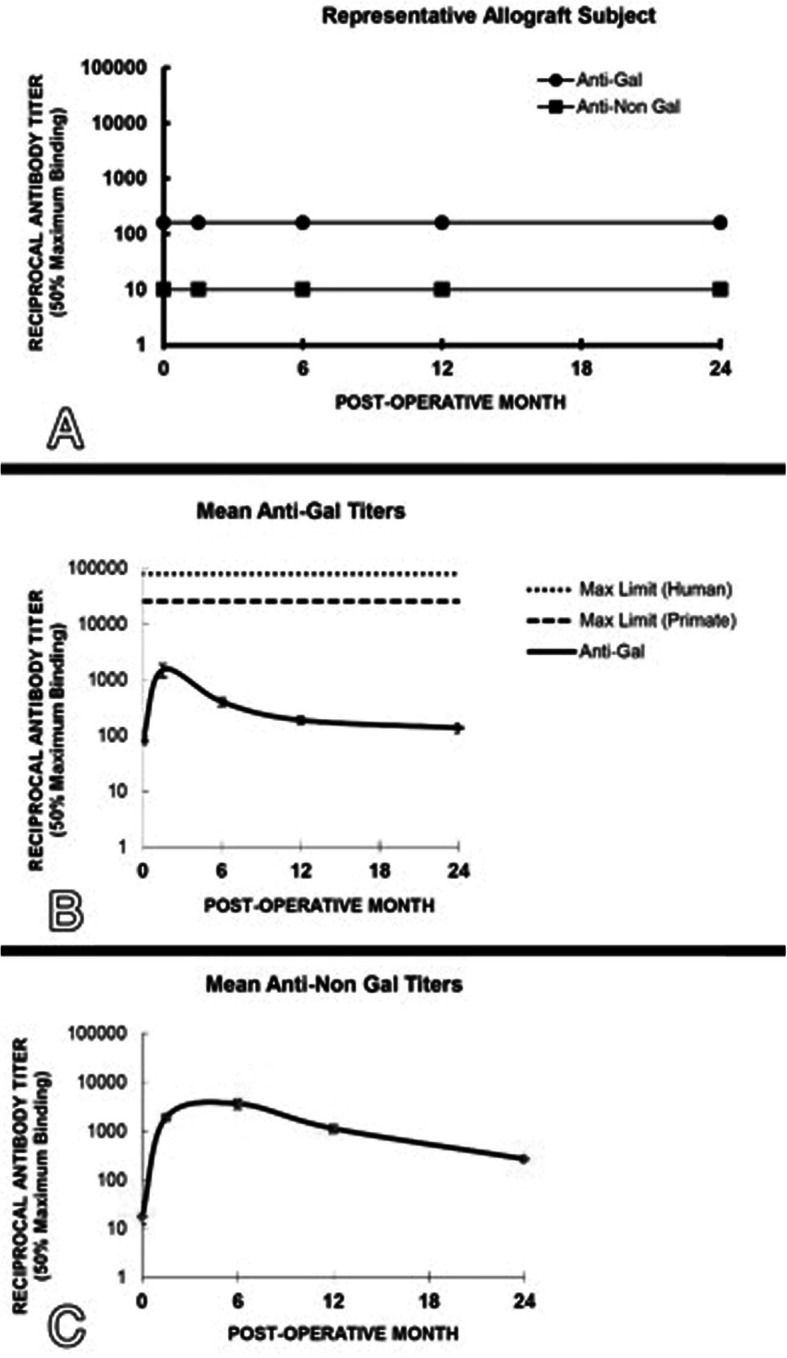
anti-Gal and anti-non Gal antibody testing results in a representative allograft patient (**a**), mean anti-Gal titers (± standard error) for the patients implanted with the xenograft device (**b**) and the anti-non Gal antibody response against the multiple immunogenic porcine proteins within the xenograft (**c**). See text for details

Figure 6a displays the anti-Gal and anti-non Gal antibody testing results in a representative allograft patient. This data is consistent with the data from the entire allograft group (data not shown), and shows that allograft recipients did not display significant anti-Gal and anti-non Gal antibody responses since the allograft implant lacks α-gal epitopes and its proteins are not immunogenic as they are of human origin.

Figure 6b shows mean anti-Gal titers (± standard error) for the patients implanted with the xenograft device. Mean anti-Gal antibody titers peak at approximately 2 months and trend lower by 6 months post-operatively. By 12 and 24 months post-operatively, the mean anti-Gal response returned nearly to baseline.

Figure 6c describes the anti-non Gal antibody response against the multiple immunogenic porcine proteins within the xenograft. Anti-non Gal response in xenograft recipients plateaued between six weeks and six months. Anti-non Gal antibody response gradually decreases in the period between the 1 and 2 year time points.

No evidence of radiological (X-ray/MRI) and serological effect of deleterious immunoreactivity (e.g., early resorption, acute humoral and local immunological mediated rejection) was seen in any of the xenograft or allograft patients, including those experienced complications or received contaminated grafts.

### Failures and complications

Six xenograft subjects received *Ralstonia pickettii* contaminated grafts. These subjects were treated with graft removal and antibiotic therapy based on the antibiogram. The unusual contamination occurred during the graft manufacturing process. *Ralstonia pickettii* is a waterborne organism that creates biofilms associated with membranes commonly found in high purity water systems. The small size of *Ralstonia pickettii* makes it a sentinel organism to challenge 0.2 μm aqueous filters. The combination of water source and the use of 0.2 μm filters in the sponsor’s manufacturing water supply was determined to be the root cause of the contamination. Corrective actions implemented by the sponsor included installation and validation of a water for injection quality purification system with 0.05 μm filters and additional decontamination procedures during tissue procurement and monitoring procedures during processing. Nonetheless, all treated xenograft devices may have been exposed. The grafts contaminated with *Ralstonia pickettii* are not representative of final product. They were considered early failures.

Overall, there were 17 serious adverse events (SAEs) in 11 subjects (Table 2). Twelve SAEs occurred in the 6 subjects who were implanted with contaminated grafts. Excepting these events, 5 SAEs were reported in 5 subjects; with 1 event in 1 subject in the allograft group (an unrelated myocardial infarction, subject 7I) and 4 events in the xenograft group, with 2 device related events in 2 subjects (persistent effusion and pain, subject 2AE; persistent pain, subject 7D) and 2 non-device related events in 2 subjects (surgical site stitch abscess, subject 2F; traumatic ACL re-rupture, subject 7E).

## Discussion

The most important finding of the present study was that the use of xenograft in ACL reconstruction was associated to high infection rate. Six of the subjects in the xenograft group (20.6%) were considered early failures, attributed to a water-based pathogen contamination in processing (manufacturing error).

The primary finding of the present study was that ITT analysis (missing/contaminated graft subjects included using the LOCF imputation method) revealed significant higher knee laxity values in xenograft group at 24-month follow-up, but subjective outcomes using indices of pain together with activity and quality of life showed inter-group comparable results. On the other side, both groups of patients achieved comparable outcomes at 24-month follow-up when per-protocol analysis was performed (missing/contaminated graft subjects excluded).

The normal temporal sequence of graft condensation, as evaluated by cross-sectional area on MRI, is seen over the 12 month to 24 month interval in both allograft and xenograft groups. This finding confirms that ligamentization and graft collagen remodeling occurs in the xenograft ACL recontruction device. An increased tunnel widening was found for xenograft implants, even if it was within the expected range of 20–35% as reported in the literature [4, 19]. Tunnel widening has been addressed in multiple peer-reviewed publications [20, 27, 28]. Although some studies suggest a relationship between widening and laxity, most authors report no association between widening and either laxity or clinical outcomes. Mirroring most reported findings, the present study did not find substantive correlation of tunnel changes with laxity or clinical performance measures in either the allograft or xenograft groups. This observation should continue to be monitored in any longer-term follow-ups.

No significant immunological reactivity was associated to xenograft group using laboratory evaluations including: immunological, white cell count, ESR, and C-reactive protein assessments. Specifically, antibody titers monitored for the galactosyl epitope and other pig epitopes were transient and considered sub-clinical. This is consistent with the previous studies and it was the result of gradual elimination of the immunogenic porcine tissue and its replacement with the recipient’s human ligament tissue [22, 25]. Anti-Gal and anti-non Gal antibody titers measured in patients who experienced complications or received contaminated grafts were not different from measurements from the rest of the xenograft cohort, consistent with the conclusion that no adverse events were the result of acute rejection of the graft or a graft versus host response.

Unlike other xenograft devices used in reconstructive surgery, this xenograft ACL reconstruction device was designed to perform biologically in a manner similar to allograft. Removal of α-gal epitopes, coupled with low-level cross-linking, attenuates immunologic recognition yet allows the biological ligamentization response that is critical to the long-term durability of the graft to proceed. This ligamentization process eventually removes all porcine tissue, as correlated by antibody response from the serological data, and remodels it with autologous ACL tissue. This dynamic, ongoing remodeling activity in the bio-implant, incorporated into the device by design, contrasts markedly with the finite duty cycle and material fatigue failure evidenced by fully cross-linked tissue grafts, such as heart valves. The operative technique and presentation of the xenograft is identical to cadaveric allografts.

Considering results of per-protocol analysis (missing/contaminated graft subjects excluded) both allografts and xenografts provide the requisite strength upon implantation [12] to support stable knee function, while also serving as a scaffold for cellular repopulation and gradual remodeling known as ligamentization [2, 8]. Allografts are associated with a very small but important risk of disease transmission as well as higher risk of graft failure for young athletically active patients [6, 7]. The variability of allograft donor tissue and resultant limitations on the availability of high quality allografts is also a concern. Age older than 40 years, and especially older than 65 years, negatively impacted biomechanical properties, whereas gender had minimal effect on the properties of allograft tissue [18]. The immuno-chemically modified xenograft investigated in this study was designed to be a direct replacement for homologous cadaveric tissue. Xenograft performance as assessed objectively and subjectively compares favorably to the concurrent allograft control as well as to published clinical experience with both autograft and allograft [5, 11, 17, 21].

### This study has some limitations

High infection rate (20.6%) was reported in xenograft group. Both groups of patients achieved comparable outcomes if missing/contaminated subjects are excluded. However; the level of water borne contamination within the clinical production lots is unknown, and some, if not all, xenografts were affected by the anomaly. With corrective actions performed to ensure manufacturing anomalies are corrected, the xenograft may perform better than the results seen within the ITT analysis (as demonstrated by the per protocol perfomed analysis). This must be considered in future studies involving xenograft for ACL reconstruction in humans.

The study was designed as a non-inferiority study with 7 surgical sites and a total enrollment of 66 (34 allograft, 32 xenograft); 28 subjects were required in each group for full statistical powering of the study. Having seven surgical sites with an average 9 subjects per site presents additional variability, considering inter-site differences in surgical technique and allograft procurement: this is another limitation (see Additional file 1 for details).

In the present work we compare xenograft to the standard of care (allograft). In different part of the world allografts were processed in different ways: this is another limitation.

## Conclusion

The present study of xenograft usage for ACL reconstruction was minimized by a high infection rate (20.6%) for xenograft receiving patients due to waterborne processing infection resulting in graft removal for these patients. The remaining xenograft patients achieved comparable clinical outcomes regarding knee stability and subjective outcomes.

Improved harvesting/processing treatments in future studies using xenografts for ACL reconstruction are needed to reduce infection rate, otherwise xenograft should not be used in ACL reconstruction.

## Supplementary information

**Additional file 1.**

**Additional file 2.**
