# Supplementary material for: Xenograft for anterior cruciate ligament reconstruction was associated with high graft processing infection
Source: J Exp Orthop. 2020 Oct 7;7:79. doi: 10.1186/s40634-020-00292-0 (PMC7541808; doi:10.1186/s40634-020-00292-0)
Supplement: Supplementary file 1 — Additional file 1. [file 40634_2020_292_MOESM1_ESM.docx]

**Appendix 1: Study Limitations**

**Surgical Technique**

|  |  | Overall | Allograft | Xenograft |
| --- | --- | --- | --- | --- |
| Transtibial | | 22% | 24% | 22% |
| Anatomic (AM) | | 78% | 76% | 78% |

**Fixation Device Materials (Interference Screws) Used, Overall and by Location**

|  | Overall | | Femoral | | Tibial | |
| --- | --- | --- | --- | --- | --- | --- |
|  | Femoral | Tibial | Allograft | Xenograft | Allograft | Xenograft |
| Metal | 64% | 15% | 33% | 30% | 8% | 8% |
| PEEK |  | 38% |  |  | 20% | 18% |
| PLLA/HA | 36% | 47% | 18% | 18% | 24% | 23% |

**Allograft Treatment**

|  |  |  | Allograft | Xenograft |
| --- | --- | --- | --- | --- |
| Irradiated (Gamma or e-Beam) | | | 31% | 100% |
| Aseptic |  |  | 69% | 0% |
