# Supplementary material for: Xenograft for anterior cruciate ligament reconstruction was associated with high graft processing infection
Source: J Exp Orthop. 2020 Oct 7;7:79. doi: 10.1186/s40634-020-00292-0 (PMC7541808; doi:10.1186/s40634-020-00292-0)
Supplement: Supplementary file 2 — Additional file 2. [file 40634_2020_292_MOESM2_ESM.doc]

**Appendix 2A. MRI Evaluation (Bone Edema adjacent to tunnels and Cartilage and Meniscal Findings) - Missing/Contaminated Graft Subjects Included (LOCF)**

|  | 1 Week | | |  | 12 Months | | |  | 24 Months | | |
| --- | --- | --- | --- | --- | --- | --- | --- | --- | --- | --- | --- |
|  | Allo  n=29 | Xeno  N=31 | p-value |  | Allo  n=27 | Xeno  n=25 | p-value |  | Allo  n=26 | Xeno  n=22 | p-value |
| No edema | 13.8% | 9.7% | 0.230 |  | 77.8% | 52.0% | 0.123 |  | 69.2% | 45.5% | 0.207 |
| Edema ≤1cm | 48.3% | 29.0% |  |  | 11.1% | 16.0% |  |  | 26.9% | 50.0% |  |
| Edema >1cm | 37.9% | 61.3% |  |  | 11.1% | 32.0% |  |  | 3.8% | 4.5% |  |
|  |  |  |  |  |  |  |  |  |  |  |  |
| Meniscal tears | 78.1% | 83.9% |  |  | 71.9% | 96.2% |  |  | 76.7% | 91.3% |  |
| Loose bodies | 0.0% | 0.0% |  |  | 6.3% | 3.8% |  |  | 10.0% | 8.7% |  |
| Changes in articular  cartilage injury | 3.1% | 0.0% |  |  | 9.4% | 3.8% |  |  | 20.0% | 13.0% |  |
| Other | 0.0% | 3.2% |  |  | 0.0% | 7.7% |  |  | 3.3% | 13.0% |  |

**Appendix 2B. MRI Evaluation (Bone Edema adjacent to tunnels and Cartilage and Meniscal Findings) - Missing/Contaminated Graft Subjects Excluded.**

|  | 1 Week | | |  | 12 Months | | |  | 24 Months | | |
| --- | --- | --- | --- | --- | --- | --- | --- | --- | --- | --- | --- |
|  | Allo  n=29 | Xeno  n=25 | p-value |  | Allo  n=27 | Xeno  n=22 | p-value |  | Allo  n=26 | Xeno  n=21 | p-value |
| No edema | 13.8% | 12.0% | 0.143 |  | 77.8% | 54.5% | 0.241 |  | 69.2% | 47.6% | 0.322 |
| Edema ≤1cm | 48.3% | 24.0% |  |  | 11.1% | 18.2% |  |  | 26.9% | 47.6% |  |
| Edema >1cm | 37.9% | 64.0% |  |  | 11.1% | 27.3% |  |  | 3.8% | 4.8% |  |
|  |  |  |  |  |  |  |  |  |  |  |  |
| Meniscal tears | 78.1% | 88.0% |  |  | 71.9% | 95.7% |  |  | 76.7% | 90.9% |  |
| Loose bodies | 0.0% | 0.0% |  |  | 6.3% | 0.0% |  |  | 10.0% | 4.5% |  |
| Changes in articular  cartilage injury | 3.1% | 0.0% |  |  | 9.4% | 4.3% |  |  | 20.0% | 13.6% |  |
| Other | 0.0% | 0.0% |  |  | 0.0% | 8.7% |  |  | 3.3% | 13.6% |  |
